# Sustaining the adequacy and competency of CDC Staff in China after COVID-19

**DOI:** 10.7189/jogh.15.03003

**Published:** 2025-02-07

**Authors:** Quan Wang, Yumeng Lv, Runzhi Han, Viroj Tangcharoensathien, Li Yang

**Affiliations:** 1 School of Public Health, Peking University, Beijing, China; 2 Saw Swee Hock School of Public Health, National University of Singapore, Singapore; 3 International Health Policy Program, Ministry of Public Health, Nonthaburi, Thailand; 4 Beijing Institute for Health Development, Peking University, Beijing, China

## Abstract

We examine the significant increase in staffing at the Chinese Center for Disease Control and Prevention (CDC) following the COVID-19 pandemic and compare it to the staffing changes post-2003 SARS outbreak. This analysis views the surge not only as compensation for long-term understaffing, but also as a response to the immediate demands of the COVID-19 crisis. We explore the implications of this increase, addressing the financial burden it imposes on the government, the challenges in maintaining a balanced human resource structure, and the potential long-term effects on public health infrastructure. Additionally, we propose strategic recommendations including reforming the income model for CDC employees, implementing strategic workforce planning, and making infrastructure improvements. These measures aim to support a more effective and resilient public health system in China.

The coronavirus disease 2019 (COVID-19) pandemic has undeniably shaped global and national pandemic preparedness, prevention, and response strategies. Lessons learned from these experiences have informed efforts to strengthen health systems resilience and minimise future risks. In China, one of the most notable responses has been the significant increase in the number of staff at the Centers for Disease Control and Prevention (CDCs), at both national and local level. This surge raised an important question: is this increase a strategic compensation for previous deficiencies or a systematic response to the unprecedented demands from the pandemic?

After the severe acute respiratory syndrome (SARS) virus outbreak in 2003, China had not experienced another large public health emergency for 17 years. During this period, there was a significant trend of gradual reduction in the number of CDC staff [1]. For instance, when China initiated the ‘new health system reform’ in 2009, the overall number of CDC staff went down by 4.6% from 197 thousand in 2009 to 188 thousand in 2019 [2]. This occurred during a period of relative calm without public health emergencies, reflecting complacency in maintaining a robust epidemiological workforce for future large outbreaks or pandemics [3].

The emergence of COVID-19 in late 2019 marked a dramatic shift in this context. The Chinese CDCs, being at the forefront of the country’s pandemic response, faced immense pressure to devise an adequate response [4]. The ‘zero COVID’ policy required an unprecedented scale of health workforce for conducting contact tracing, mass testing, quarantine enforcement, and public health communication [5], These demands exposed the gaps in the existing workforce and infrastructure, necessitating immediate and substantial increases in staffing levels, funding, and operation capacities. The number of CDC staff had reached 240 thousand in 2022, a 27.7% increase from 2019 (**Figure 1**, **Box 1**) [8].

**Figure 1:**
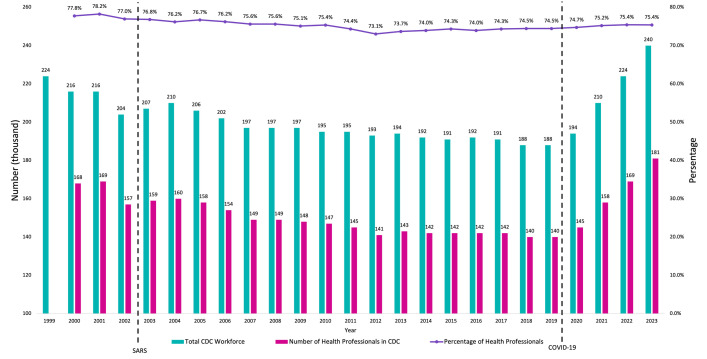
The total workforce of CDCs in China (1999–2023), determined based on China Statistical Yearbooks published between 2000 and 2024.

Box 1 **The evolution and establishment of Chinese CDCs**

China's CDC system has undergone significant reforms since the SARS outbreak in 2003. The Health Supervision Institute took over the functions of law enforcement and health supervision, while the CDC became a technical support department. Currently, China’s CDC system comprises a four-level structure: national, provincial, municipal, and county [6]. The CDC itself has been integrated with and responsible by three levels of local government. While the higher-level (national) CDC does not have direct management authority over the lower-level (provincial, municipal and county) CDCs, it provides technical guidance and support. The sub-national level CDC operates as a subordinate agency of local governments that directly manage, finance, and organise it to ensure the effective implementation of local public health interventions [7].

Internationally, there is substantial research on the impact of COVID-19 on health workers at the individual level. A systematic review and meta-analysis found an increase in burnout and mental health problems among health workers at the frontline and in healthcare facilities providing clinical services for severe cases during the surge of the COVID-19 pandemic, reporting high pooled prevalences for burnout (43.6%), anxiety (37.1%), depression (37.6%), insomnia (43.7%), stress (41.3%), posttraumatic stress disorder symptoms (30.6%), and somatic symptoms (25.0%) [9]. Effective interventions to address psychosocial consequences on the healthcare workforce during and after the pandemic have been available and need to be scaled up [10]. The Joint External Evaluation of the World Health Organization defines an optimal target for attaining and complying with the International Health Regulation (2005) as one trained field epidemiologist or equivalent per 200 000 population [11].

To our knowledge, however, there have been no assessments of the changes in the number of disease control staff at a national level before and during the pandemic. This viewpoint fills the gap and calls for future studies on the healthcare workforce supporting disease control programmes.

By the end of 2022, the government declared the end of the COVID-19 pandemic, cancelled the ‘zero COVID’ policy, and classified COVID-19 as a category II infectious disease (similar to tuberculosis, AIDS, and viral hepatitis) [12]. Consequently, the demand for a workforce to implement the ‘zero COVID’ policy was eliminated.

## COMPENSATION OR SYSTEMATIC RESPONSE?

The increase in CDC staff may be a reactive response to the surge in demand, particularly as the ‘zero-COVID’ policies created an unprecedented workload that necessitated more disease control personnel for testing, tracing, and quarantine efforts.

Numerous studies, including our long-term interaction and interviews with stakeholders, indicate that the CDC workforce attrition before 2019 was primarily due to relatively low salaries [13]. A director of the CDC in Jinan shared that, a year before the COVID-19 pandemic, his Center can only recruit two employees to fill five vacant posts. In China, disease control is considered a public service, meaning the CDC staff are not allowed to generate additional income apart from the relatively fixed government salaries. Additionally, disease control was not a priority for local governments before 2020, leading to insufficient attention and funding support [14]. Consequently, many graduates do not see disease control work as a promising career path, resulting in a long-standing shortage of CDC manpower in the national and local governments [15].

The government, however, went on to invest substantial resources in the CDC during COVID-19, thereby helping compensate the previously low-paid disease control staff. A CDC director in Beijing shared with us during an interview that the number of positions had increased from about 280 in 2019 to around 500 in 2024 – an initiative aimed at addressing the increased demand from the pandemic. Additional financial incentives provided by local governments made disease control jobs more attractive. Moreover, permanent government official positions with stable salaries and high social respect became highly valued during the pandemic compared to those in the private sector, which became sluggish due to the economic shutdown [16].

The end of the ‘zero COVID’ policy saw a significant reduction in the CDC's workload and the reversal of the additional incentives to pre-pandemic levels. The surge in the number of staff, however, led to a problem of oversupply and became a fiscal burden for the government. Based on our interviews with CDC leaders in Beijing, several local CDCs redeployed them to other functions, such as public health commissioner in rural areas, while CDC staff on temporary posts without permanent positions or full budgets saw their employment terminated.

## HISTORY REPEATS ITSELF: UPS AND DOWNS OF CDC STAFF

The substantial increase in the number of disease control staff after the COVID-19 pandemic has significant long-term implications for China’s public health infrastructure. However, it is not the first time that such a surge occurred (**Figure 1**). After the SARS outbreak in 2003, there was a modest, 3% increase in the total number of CDC staff over two years [17], followed by a decline. Similarly, COVID-19 has led to a significant and greater increase in CDC staffing, with an approximate 28% increase over three years [8]. However, with the population growth in China, there were 16.9 CDC staff per thousand population in 2023, still lower than 17.9 in 1999 [2]. However, the full use of technology during COVID-19 may have compensated for a part of the staff’s workload (**Box 2**).

Box 2 **The application of technology in disease control**

The utilisation of advanced technology in China’s CDC system has transformed disease control workload by reducing the overall demand for CDC staff. For example, China's web-based infectious disease reporting system has enabled real-time tracking and reporting of cases, minimising the need for extensive manual data collection and entry. Similarly, the health QR code system used during COVID-19 allowed for efficient contact tracing, risk assessment, and public health communication, reaching millions with minimal staff requirement. These technologies, however, require personnel with advanced technical skills who can operate and maintain them, as well as analyse data effectively. Though the application of IT has reduced the reliance on large numbers of field staff, it increased the demand for a more specialised, technically-proficient workforce capable of leveraging these digital tools to enhance CDC’s efficiency and responsiveness.

Sustaining these levels can be challenging without long-term structural reforms. The Chinese government has not reformed the basic salary structure for CDC employees to be more attractive. The additional workload-related payment was cancelled after the pandemic. As the economy gradually improves, better-paid jobs in private enterprises or hospitals become more attractive, resulting in staff leaving the CDC for more lucrative opportunities and bringing their numbers down to the level of 2019 pre-pandemic period. This potential attrition could undermine the capacity to prepare and respond to a future public health emergency.

The rapid growth of the workforce also affected the relatively stable human resource structure, including the age structure and number of positions available higher up in the hierarchy. The number of CDC employees increased by about 20% in three years during COVID-19; due to China's campus recruitment policy, these new employees were often recent college graduates. This influx created significant pressure for career promotions, as the quota for higher positions and technical titles is relatively fixed, making this career path less attractive for new employees. The existing infrastructure at the CDC also needs to be upgraded rapidly; some Centers are already facing issues such as insufficient office space, forcing them to send new employees to work in community settings. Moreover, the addition of a large number of employees in a short period means that a similar number will retire around the same time in the future, which will likely disrupt the stability of disease control operations, presenting significant challenges for maintaining continuity and effectiveness in public health efforts.

In addition, the proportion of health professionals in the overall number of CDC employees has remained basically unchanged, at around 75% to 76% (N) [8], showing that, while the CDCs’ manpower has increased significantly during the pandemic, the total number of administrative staff has also increased simultaneously. Considering that the CDC is essentially a technical department, the increase in non-technical personnel does not strengthen the scientific and technical expertise necessary for effective disease control.

## HOW CAN WE MAKE A DIFFERENCE?

Nevertheless, this increase in CDC staff will help further strengthen China's disease control work. A comprehensive reform of the remuneration structure for CDC employees is essential for achieving this. Ensuring competitive salaries and benefit packages, including performance-based incentives, fellowship for postgraduate education, and in-service training, can help retain talent and foster staff competency in the organisation, as well as attract new graduates [1].

Strategic workforce planning is also crucial here [18]. There is a need to estimate the size of core staff required outside of public health crises; by different levels of local government and population size; for conducting local disease outbreak surveillance, investigation, and control; and for maintaining laboratory capacity. An additional workforce should also be established to be deployed rapidly given small-, medium-, and large-sized public health emergencies. This requires regular table-top exercise to keep systems alert and well prepared. Application and full use of technology can replace part of staffs’ workload and improve efficiency.

While the primary function of the CDC is often associated with controlling communicable diseases, addressing non-communicable diseases (NCDs) is equally crucial. In 2019, nine out of the top ten causes of disability-adjusted life year losses in China were attributable to NCDs, with lung cancer, ischaemic heart disease, and diabetes showing the largest increases between 2009 and 2019 [19]. Road injuries, ranking fifth, were the only non-disease-related cause, and they have conversely declined over the same period. Given the significant health burden posed by NCDs, redeploying part of this newly-recruited CDC workforce is essential and could be achieved through reskill, re-orientation, and reassignment. This would not only optimise the utilisation of the expanded staff, but also strengthen China's public health response to its leading NCD challenges, ensuring a more comprehensive approach to disease prevention and health promotion.

It is also vital to review and adjust the proportion of administrative and technical staff within the CDC to maintain its core technical functions. The newly established Disease Control and Prevention Administration presents a significant opportunity in this regard. As an administrative department, it can absorb part of the CDC's administrative staff, allowing it to focus more on its core technical and scientific functions.

## CONCLUSIONS

The substantial increase in CDC staffing since the COVID-19 pandemic has brought both opportunities and challenges to China's public health policy. While the surge has strengthened the CDC’s capacity to respond to COVID-19, we recommend further reform of the incentive structure, additional strategic planning to maintain the size core capacity outside of public health emergencies, and the establishment of a dedicated workforce that would be deployable during such crises.
